# A SFTD Algorithm for Optimizing the Performance of the Readout Strategy of Residence Time Difference Fluxgate

**DOI:** 10.3390/s18113985

**Published:** 2018-11-16

**Authors:** Siyu Chen, Yanzhang Wang, Jun Lin

**Affiliations:** 1 Key Laboratory of Geophysical Exploration Equipment, Ministry of Education (Jilin University), Changchun 130026, China; sychen15@mails.jlu.edu.cn (S.C.); lin_jun@jlu.edu.cn (J.L.); 2 College of Instrumentation and Electrical Engineering, Jilin University, Changchun 130021, China

**Keywords:** time–frequency transformation, RTD fluxgate, noise reduction algorithm

## Abstract

Residence time difference (RTD) fluxgate sensor is a potential device to measure the DC or low-frequency magnetic field in the time domain. Nevertheless, jitter noise and magnetic noise severely affect the detection result. A novel post-processing algorithm for jitter noise reduction of RTD fluxgate output strategy based on the single-frequency time difference (SFTD) method is proposed in this study to boost the performance of the RTD system. This algorithm extracts the signal that has a fixed frequency and preserves its time-domain information via a time–frequency transformation method. Thereby, the single-frequency signal without jitter noise, which still contains the ambient field information in its time difference, is yielded. Consequently, compared with the traditional comparator RTD method (CRTD), the stability of the RTD estimation (in other words, the signal-to-noise ratio of residence time difference) has been significantly boosted with sensitivity of 4.3 μs/nT. Furthermore, the experimental results reveal that the RTD fluxgate is comparable to harmonic fluxgate sensors, in terms of noise floor.

## 1. Introduction

Fluxgate is a suitable vector magnetic field sensor for field detection [1], such as nondestructive tests [2], attitude correction, and marine magnetic survey [3]. High temperature superconducting Quantum interference devices (SQUID) and giant magnetoresistance (GMR) sensors are usually considered competitors. The former offers a higher noise sensitivity of (0.1–1) pT/√Hz but with a high cost, as well as difficult operating conditions, and the latter has much in common with fluxgate but has a more complicated structure of magnetic core. Fluxgate systems prevail over these competitive technologies on account of their portable structure, simple operating conditions, and remarkable thermal and long-term stability [4]. Residence time difference (RTD) fluxgate sensors, as a new type of fluxgate magnetometers, are of interest to sense weak magnetic fields, not merely because of a low power consumption of 1 mW (usually a dozen mW of traditional fluxgates), but because of its simple signal processing circuit and intrinsic digital readout strategy [5]. These advantages can make it more suitable to severe environments.

The magnetic field information is contained in the time difference between the positive and negative saturated states. Therefore, the jitter noise superimposed on the induced signal in the time domain restricts its development. RTD fluxgates typically have the resolution of few nanotesla [6,7], which decreases competitiveness compared with the traditional harmonic fluxgate with a resolution of 10 pT [8]. Consequently, an effective method for noise reduction in the output strategy of an RTD fluxgate is imperative.

An efficient way to suppress the noise floor of the RTD output strategy and improve the resolution of the RTD fluxgate is to optimize the post-processing method of the RTD fluxgate. Hence, many contributors have conducted relevant research [7,9,10,11,12,13]. Andò et al. presented a data-post-processing strategy of RTD fluxgate sensors called “central peak time” [7]. The method reduces the estimation error of the time difference by fixing the initial time point and then utilizes an internal reference time (*t*_0_) as the timing zero point and the middle peak instant (*t_pk_*) of the spiking output as the terminal point. The target field information is contained in the difference between *t*_0_ and *t_pk_*. Although the method reduces the fluctuation in the time differences, it sacrifices the sensitivity of the instrument under the same excitation condition. The resolution is slightly improved compared with the traditional RTD detection method. Liu et al. proposed a signal detection method of RTD fluxgate sensor [9,10], which can be divided into three steps, namely, (a) converting the spiking output of RTD fluxgate into a digital signal through an analog–digital converter, (b) performing data fitting on the successive peak of the signal, and (c) calculating the time difference with time instants at the site of the three-consecutive maximum of the fitted signal. This method immunizes the fluctuation in time differences from the threshold of the comparator, with which the detection precision of the sensor has been improved. Nevertheless, the experimental sensitivity of the fluxgate is proved to be 0.0085 μs/nT. A new post-processing algorithm that can diminish the fluctuation in the RTD and maintain the sensitivity at the same time should be conceived to optimize the performance of RTD fluxgate magnetometers authentically.

This paper presents a signal post-processing method combined with frequency domain, which can remedy the shortcomings of the signal detection method in previous methods of the RTD fluxgate sensor. This method utilizes the time–frequency transformation, which extracts the time information of a single frequency of the induced voltage signal. The experimental sensitivity is maintained as in the traditional comparator RTD (CRTD) method. The stability of time difference is boosted significantly; the method would not be subject to threshold interference.

## 2. Basic Theory and Noise Sources of Residence Time Difference Magnetometer

**Figure 1:**
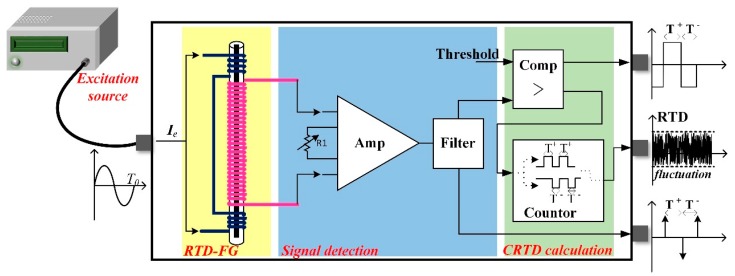
Block diagram of the traditional residence time difference (RTD) fluxgate sensor system and the working principle of the traditional RTD fluxgate system with the comparator RTD (CRTD) method.

The working principle of traditional RTD fluxgate is introduced briefly. As shown in Figure 1, the RTD fluxgate system is composed of an excitation current source, a magnetic sensor with Co-based amorphous material and two windings (a primary winding and a secondary winding), the signal detection circuit, and the RTD calculation module. The time difference estimation method in the RTD system is implemented by the hardware, which is called the CRTD method for distinguishing the single-frequency time difference (SFTD) method presented in this paper.

The spiking output is transformed into a square signal via a Schmitt trigger. The positive saturation time, which is labeled $T^{+}$, and the negative saturation time, which is labeled $T^{-}$, are counted separately. The time difference between $T^{+}$ and $T^{-}$ contains the target magnetic field information. The mathematical expression of time difference and the sensitivity of the RTD magnetometer are shown as Equation (1) and Equation (2), respectively [7]. The excitation signal is selected as a sinusoidal signal throughout this paper [14,15],

(1) $$RTD=T^{+}-T^{-}=\frac{2}{\omega }\left[\arcsin\left(\frac{H_{c}+H_{x}}{H_{e}}\right)-\arcsin\left(\frac{H_{c}-H_{x}}{H_{e}}\right)\right],$$

(2) $$S=\frac{\partial RTD}{\partial H_{x}}=\frac{2}{\omega }\left[\frac{1}{\sqrt{H_{e}^{2}-{\left(H_{c}+H_{x}\right)}^{2}}}+\frac{1}{\sqrt{H_{e}^{2}-{\left(H_{c}-H_{x}\right)}^{2}}}\right],$$

where $H_{c}$ denotes the coercivity of soft magnetic material; $H_{e}$ and ω denote the amplitude and angular frequency of the driving field, respectively; and $H_{x}$ denotes the target magnetic field along the axis direction of the magnetic core.

The above expressions show that the sensitivity of RTD fluxgate is only related to the driving conditions (supposing the coercivity and target magnetic field as constant) and inversely proportional to the driving conditions, including the amplitude and frequency of the excitation source. High sensitivity and low fluctuation guarantee low noise floor of the RTD fluxgate. However, the sensitivity and fluctuation cannot be optimized at the same time. If the driving conditions are decreased for a high-sensitivity performance, the fluctuation of RTD will increase as well. Therefore, reducing only the excitation conditions is not the solution for obtaining an optimized performance of RTD magnetometer.

**Figure 2:**
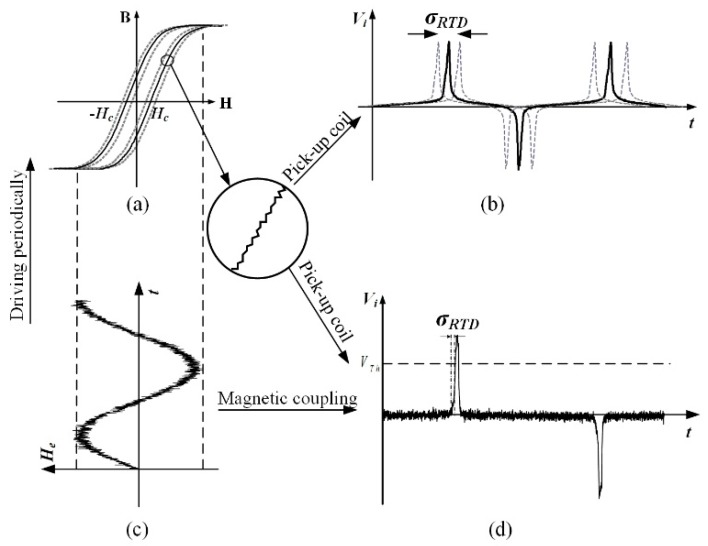
The uncertainty in RTD estimation. (**a**) Hysteresis loop of the magnetic core with repeatability error; (**b**) induced voltage signal with vibration error; (**c**) an excitation signal superimposed with jitter noise; (**d**) induced signal superimposed with jitter noise.

The qualitative description of the RTD noise effect was analyzed by Andό et al. [5]. Some possible solutions, such as shielding, bias field filtering, and increasing the slope of the excitation signal to suppress the noise were presented in the paper. The fluctuation in RTD, as depicted in Figure 2, is compactly related to the “magnetic” noise, which is modeled as a fluctuation of coercivity $H_{c}$ (in Figure 2a), and the “electrical” noise superimposed on the output signal (in Figure 2d). In the current study, the error of RTD estimation that stems from the magnetic core and the excitation signal is divided into two aspects. First, the repetitive error of the hysteresis loop that shows as the instability of the coercive force *H_c_* and high-frequency electronic noise superimposed on the excitation signal can cause vibration deviation [16]. Second, the discontinuity of magnetization (Barkhausen noise) and weak electromagnetic coupling between primary and secondary coils (this is also the reason why lowering only the excitation signal is not the solution for increasing the sensitivity) can cause jitter noise introduced on the inductive signal, named the jitter deviation [17]. $\sigma_{vib}$ and $\sigma_{jitter}$ denote the estimation uncertainty of RTD caused by the vibration noise (including the deviation owing to the repetitive error of coercivity $\sigma_{vib-coe}$ and the local fluctuation of bias signal $\sigma_{vib-thr}$) and the jitter noise, respectively. The excitation signal is considered ideal in this paper. Therefore, the true value of RTD should add the deviations that are affected by $\sigma_{vib-coe}$ and $\sigma_{jitter}$, and the expression of the time differences in the CRTD method is shown as Equations (3) and (4).

(3) $$\sigma_{CRTD}=\sqrt{\sigma_{vib-coe}^{2}+\sigma_{jitter}^{2}},$$

(4) $$RTD_{CRTD}=RTD+\sigma_{CRTD}.$$

**Figure 3:**
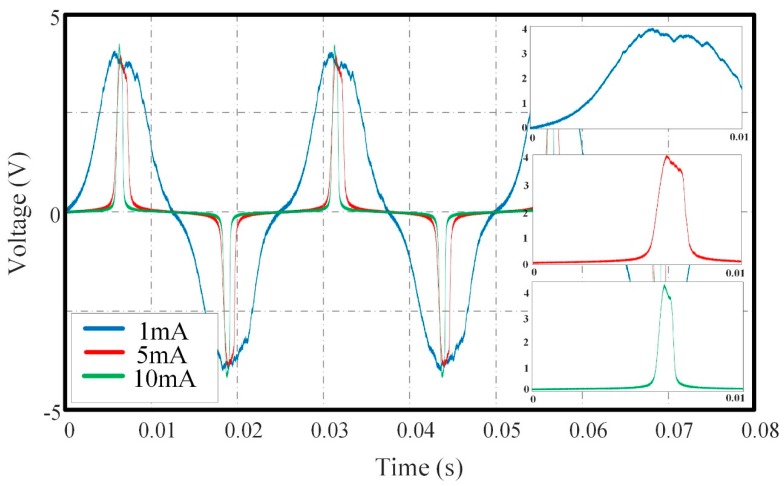
Induced voltage of RTD fluxgate sensor with excitation frequency of 40 Hz and different excitation amplitude of 1 mA, 5 mA and 10 mA.

Induced voltage signals in different conditions of excitation amplitude are shown in Figure 3. Some conclusions can be obtained: (a) The jitter noise is superimposed on the induced signal, and the amplitude of excitation signal influences the shape of induced voltage at peak value position. (b) The slope of increasing part decreases as the excitation amplitude declines, which leads to a larger estimation error of RTD. Contraposing this phenomenon, the algorithm addressed in this paper can suppress the jitter noise to a great extent and make the induced signal at peak value position smooth.

## 3. Single Frequency Time Difference Method

The amplitude *H_e_* of cosine excitation signal with period *T*_0_ drives the magnetic core to a bistable saturate state, and the induced electromotive force e(t), which is shown in Equation (5) and Equation (6), is obtained via Faraday’s law of electromagnetic induction,

(5) $$e\left(t\right)=-N_{r}S_{r}\frac{dB}{dH}\cdot \frac{dH}{dt}+N_{jitter}\left(t\right),$$

(6) $$H\left(t\right)=H_{e}\left(t\right)\pm H_{x},$$

where $N_{r}$ refers to the turns of the induced coil, and $S_{r}$ is the valid cross-sectional area of the magnetic core. $N_{jitter}\left(t\right)$ is the jitter noise superimposed to the induced output due to the magnetic noise and electronic noise, and its distribution characteristic obeys Gaussian distribution [18]. H(t) is relative to two factors at least, which are the driving magnetic signal $H_{e}\left(t\right)$ and the ambient magnetic field $H_{x}$. The two factors throughout this paper are assumed to be parallel. In addition, the total field is considered homogeneous within the core volume.

The induced output is a consecutive series of positive and negative spiking signal with the same period *T*_0_ as excitation signal based on the effect of electromagnetic couple effect. However, $\mu_{r}\left(t\right)$ is a complicated function as a consequence of the nonlinear relationship between magnetic field intensity *H* and magnetic induction intensity *B*. For simplifying the proceeding of analysis, the squareness ratio of hysteresis loop of the magnetic core is assumed to be 1, that is, the transition time between positive and negative states is instantaneous [19,20]. The assumption is reasonable because the transition time is generally negligible compared with the period of the excitation signal.

An analytical model of the fluxgate magnetometers can be found in [11], and the sensor response can be described as a series of voltage pulses. Hence, the response of the fluxgate sensor shown in Equation (5) can be rewritten as Equation (7),

(7) $$e\left(t\right)=V_{p,m}\left(t\right)=C\cdot {\left(-1\right)}^{m}\cdot \mu_{m}\left(t\right)\cdot f_{m}\left(t\right)\cdot \delta \left[t-T\left(m\right)\right]+N_{jitter}\left(t\right),$$

(8) $$\mu \left(t\right)=\frac{dB}{dH},\;f\left(t\right)=\frac{dH}{dt},$$

(9) $$T\left(m\right)=H_{e,m}{}^{-1}\left(-H_{x}+{\left(-1\right)}^{m-1}\cdot H_{c,m}\right),$$

where $C=-S_{r}\cdot N_{r}$ is a constant, and *m* is the ordinal number of the current voltage pulse. $H_{e,m}{}^{-1}$ is the inverse function of the excitation signal. However, there is some differences from [11] that are worth being noted. Firstly, the induced voltage *e*(*t*) is no longer the periodic extension of *V_p,m_*(*t*) because of the electric noise and magnetic noise mentioned above. Secondly, T(m) the time instant that the voltage pulse appears will be affected by the both noise, that is $T\left(m\right)=T+\sigma_{vib-coe,m}$, where *T* denotes the time instant calculated in [11] within one period.

The operator short-time Fourier transform (STFT) is utilized for extracting the single-frequency signal of the RTD response. The basis function is the main distinction between the STFT and Fourier transform (FT), which is $e^{-j\omega t}$ in FT substituted by $\gamma \left(\tau -t\right)\cdot e^{-j\omega \tau }$ in STFT. The window function γ(t) offers time information in a frequency domain. Therefore, the RTD can be obtained from a single-frequency signal. The time–frequency transformation of the induced voltage signal is shown as

(10) $$E\left(t,\omega \right)=STFT\left[e\left(t\right)\right]={STFT\left[V_{p,m}\left(t\right)\right]+STFT\left[N_{jitter}\left(t\right)\right]|}_{\omega =\omega_{0}}.$$

The formulation can be rewritten as Equation (11) by Equation (10) into Equation (7).

(11) $$E\left(t,\omega \right)=C\cdot {\left(-1\right)}^{m}\cdot STFT\left[\delta \left(t-T\left(m\right)\right)\right]⊗STFT\left[\mu_{m}\left(t\right)\right]⊗STFT\left[f_{m}\left(t\right)\right],$$

(12) $$STFT\left[x\left(t\right)\right]=\int_{-t_{\gamma }/2}^{t_{\gamma }/2}x\left(\tau \right)\gamma \left(\tau -t\right)e^{-j\omega \tau }d\tau .$$

**Figure 4:**
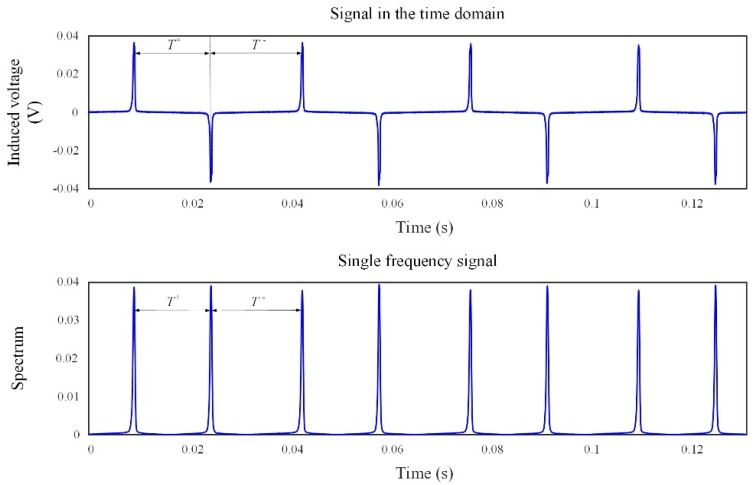
Output spiking signal in the time domain (upper, @30 Hz) and time–frequency transformation of the output spiking signal (nether, $f=f_{0}=30\;\mathrm{Hz}$).

**Figure 5:**
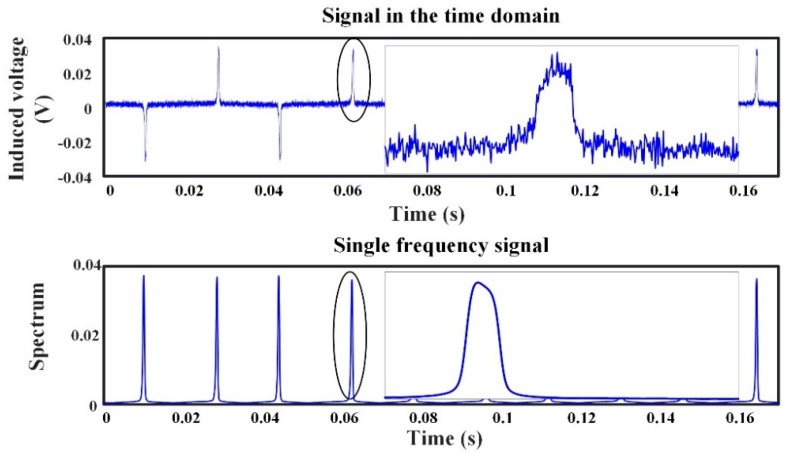
Induced signal in the time domain (upper, @30 Hz) versus single-frequency signal (nether, $f=f_{0}=30\;\mathrm{Hz}$); zoom in on the horizontal axis only.

In Equation (12), γ(τ−t) is the window function to truncate the calculated signal x(t), and $t_{\gamma }$ is the length of the window function. The signal should be discrete for further processing. The discrete time–frequency transformation of e(t) should be described as

(13) $$\begin{array}{l} E\left[a,n\right]={\left(\sum_{i=0}^{L-1}V_{p,m}\left[i\right]\cdot \gamma \left[i-a\right]W_{L}^{ni}+\sum_{i=0}^{L-1}N_{jitter}\left[i\right]\cdot \gamma \left[i-a\right]W_{L}^{ni}\right)|}_{\omega =\omega_{0}},\\ t=aT_{s},\omega =2\pi n/\left(L\cdot T_{s}\right),W_{L}=e^{-j2\pi /L}, \end{array}$$

where $T_{s}$ denotes the sample time interval; *a* and *n* are positive integers; and *L* is the length of the window function, $N_{jitter}\left[n\right]$ denotes discrete jitter noise. The variate *t* denotes the observation time, and the variate *ω* denotes the angular frequency. The variate *t* denotes the observation time, and the variate *ω* denotes the angular frequency. The frequency resolution is decided by the sample frequency and the numbers of the fast Fourier transform (FFT). The time resolution is reconstructed by the sample frequency and the length of the signal for RTD calculation. Equation (13) shows that when the frequency is fixed at a low frequency *ω*_0_, the jitter noise is significantly suppressed; thus, its value is approximately equal to zero. The result of a single-frequency signal and the comparison between induced voltage with jitter noise and the spectrum of a single-frequency signal are shown in Figure 4 and Figure 5, respectively.

Figure 4 shows that the spectrum of a single-frequency signal achieves a maximum value at the time instant when the spiking of the induced signal appears, as in the CRTD working method, which provides the essential condition for the time difference estimation. Therefore, the single-frequency signal still contains the target field information.

As shown in Figure 5, the induced signal in the time domain (diagram above, titled signal in time domain) is of obvious jitter noise (selecting the filter with a suitable cutoff frequency $f_{c}=300\;\mathrm{Hz}$ can suppress some of the broadband noise, not all of them). Figure 5 below depicts that the single-frequency signal at 30 Hz is smooth, and the jitter noise is obviously suppressed. The time–frequency transformation can thus be utilized to obtain the target field information, which is still contained in the time difference of the single-frequency signal.

The time instant at the maximum value of the transformation, denoting as *t_i_*, has to be selected to calculate RTD. The time instant is obtained within every half period, and the successive time instants should be applied to acquire an RTD. Therefore, the vibration noise will be the only impact factor. The expression of RTD is shown as Equations (14) and (15). Obviously, $\sigma_{vib-coe}$ is smaller than $\sigma_{CRTD}$ in Equation (3).

(14) $$RTD_{i}=T^{+}-T^{-}=\left(t_{i+1}-t_{i}\right)-\left(t_{i+2}-t_{i+1}\right)+\sigma_{vib-coe},$$

(15) $$RTD_{SFTD}=RTD+\sigma_{vib-coe}.$$

The uncertainty (both $\sigma_{jitter}$ and $\sigma_{vib}$) of the RTD estimation should be quantified as a few microseconds [15]. As an example, for $H_{x}=0.5pT$, the RTD would be approximately 200 μs. Thus, the deviation caused by jitter noise can be ignored. Equations (4) and (14) are combined, and the sensitivity of RTD fluxgate in CRTD and SFTD working modes are approximately equal.

## 4. Experimental Result: CRTD versus SFTD

**Figure 6:**
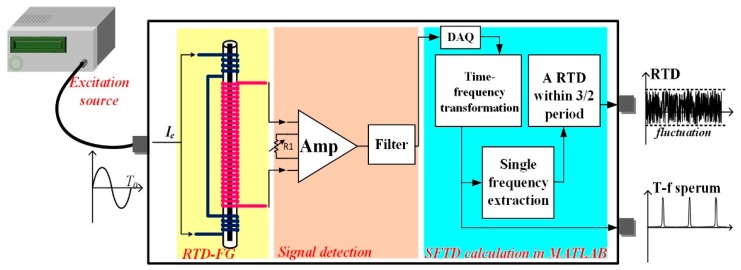
RTD fluxgate sensor system with single-frequency time difference (SFTD) method. DAQ: data acquisition.

The experimental results of the traditional system with CRTD method are compared with those of SFTD through the sensitivity and resolution of the RTD fluxgate sensor. The RTD fluxgate sensor system with the SFTD method is shown in Figure 6. The difference between the CRTD and SFTD methods is the data acquisition (DAQ) method. The SFTD method adopts software to calculate time difference, with which it can not only maintain the same effect as that in the CRTD mode but also simplify the circuit structure. The software MATLAB (MathWorks^®^, Natick, MA, USA, R2016a) is applied to estimate the time difference. A data acquisition (DAQ) module with a maximum sample frequency of 2 MHZ from National Instruments^®^ Company (in Austin, TX, USA) is applied to discretize the output induced signal. As analyzed previously, the jitter noise is suppressed significantly, and only the vibration noise affects the accuracy of the time difference estimation in SFTD.

**Figure 7:**
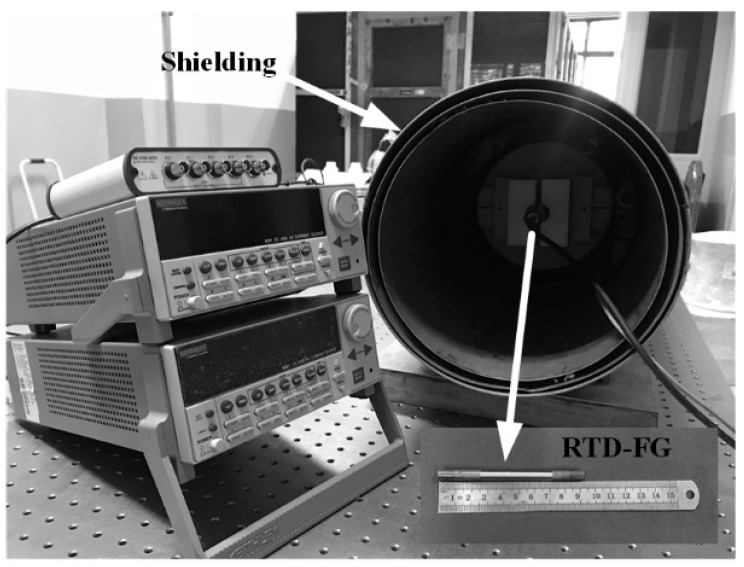
Testing system that includes RTD fluxgate, excitation signal generator, DAQ equipment, coil producing bias field and shield cylinder.

The testing system is shown in Figure 7. The magnetic core that requires a sharp hysteresis loop is an amorphous ribbon with a length of 90 mm and a width of 0.8 mm. The sensor has a two shells structure which can effectively avoid the noise caused by mechanical deformation: the inner shell is a quartz glass tube with excitation coil, and the outer shell is a plastic pipe with induced coil. Clearly shown in Figure 7, a DC magnetic field generator is placed in line with the magnetic shielding tube, which has a shielding factor of up to 98%. The frequency and amplitude of the driving signal are 20 Hz and 1 mA, respectively.

As a conclusion, the steps of the SFTD method are shown as follows.

(a) Selecting a sample frequency as high as possible and discretizing the induced voltage signal.
(b) Calculating the time–frequency transformation of the signal with Equation (12) and Equation (13) in MATLAB (The frequency resolution is equal to the sample frequency divided by the amount of points that are involved in Fourier transform).
(c) Obtaining the time instant at the maximum value of the amplitude spectrum and using the successive three time-instants to obtain *T^+^* and *T^−^*, then RTD = *T^+^* − *T^−^*.

Unlike the CRTD method, SFTD applies software calculation to extract single-frequency signals, which is equivalent to denoising the original signal and using three peak time instants in a row to estimate the time difference. Some contrastive results, such as the sensitivity of the RTD sensor, the raw data of the RTD, and the Allan variance of RTD, are shown as follows.

**Figure 8:**
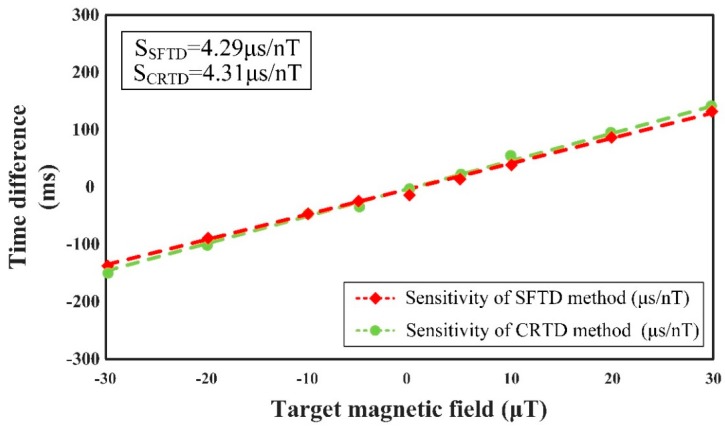
Sensitivity of RTD fluxgate sensor in different methods (SFTD method in red dashed line with rhombus markers and CRTD method in green dashed line with circular markers), in which the frequency and the amplitude of the driving signal are 20 Hz and 1 mA, respectively.

Figure 8 presents that the sensitivities of different methods are approximately the same, which are 4.29 μs/nT in the SFTD method and 4.31μs/nT in the CRTD method, through the calibration diagram of sensitivity as a function of the ambient magnetic field *H_x_* and time difference.

**Figure 9:**
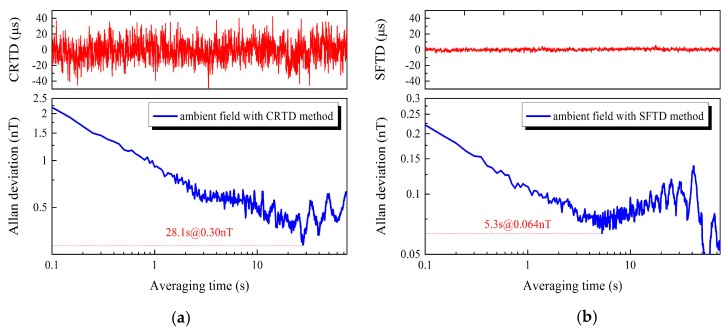
Raw RTD data and corresponding Allan deviation with different methods, (**a**) CRTD method (**b**) SFTD method, in which the excitation signal is 1 mA@20 Hz.

The experimental results shown in Figure 9 above prove that the SFTD method is valid to suppress jitter noise which stems from bad coupling and Barkhausen noise. The fluctuation of RTD is decreased to 10% of that upon adopting the CRTD method. The Allan variance method [21] is utilized to judge the stability and the resolution of the system in two operating conditions. Pictures below in Figure 9 shows an improvement of the device resolution with the detection limits of 300 and 64 pT. The “average time” of horizontal axis denotes the Allan variance varies with cumulation and averaging of time. The stability of RTD is greatly promoted on account of the initial point at 2.25 and 0.225 nT of the Allan variance for the different methods. The remarkable suppression of jitter noise is confirmed by the earlier inflection point in the SFTD (5.3 s) method, given that the white noise is dominant at the part before the first inflection point of Allan variance. Furthermore, the oscillation after inflection point is shown as a consequence of data shortage without reference significance. It is noteworthy that pictures above in Figure 9 are of unit with μs and pictures below are of unit with nT. The relationship there denotes as $H_{x}=\left(CRTD\vee SFTD\right)/Sensitivity$.

**Figure 10:**
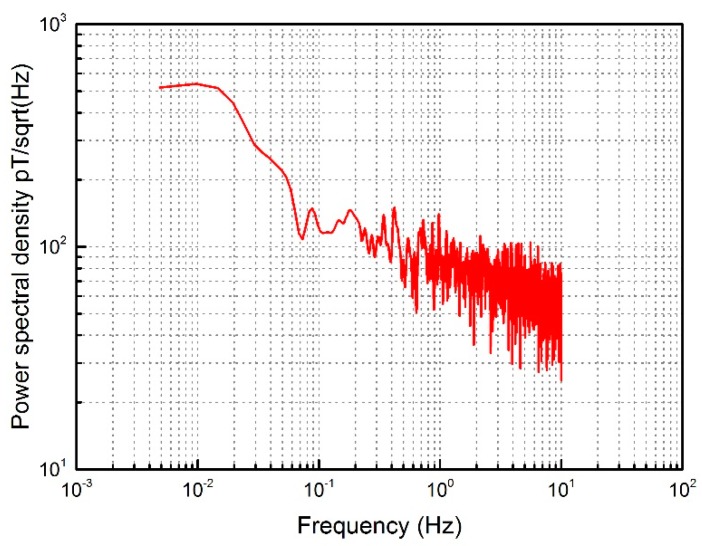
Power spectrum density of RTD with SFTD method.

The power spectrum density is utilized to calculate the noise floor of the system using the SFTD method shown in Figure 10. The noise level is 80 pT/√Hz @1 Hz, which is comparable to that of mag-03, a traditional fluxgate sensor from Bartington^®^ Instruments Company (Witney, UK). The noise floor of basic edition mag-03 is measured as 40 pT/√Hz @1 Hz under the same experimental conditions, while the sample frequency is 1 kHz.

From the promising results, the advantages of this method are concluded threefold, namely, (a) avoiding the selection of threshold value that affects the time difference estimation results, (b) simplifying the signal-conditioning circuit, and (c) optimizing the noise performance of the RTD fluxgate magnetometer.

## 5. Discussion and Conclusions

A series of potential experimental results is presented in this paper, which highlights the advantages of the SFTD method in terms of sensitivity and noise floor results. The fluctuation in RTD is caused by the vibration noise and jitter noise in the traditional RTD method. Equation (2) shows that the sensitivity of RTD fluxgate magnetometer is inversely proportional to excitation conditions. When the excitation field is small (obtaining high sensitivity), jitter noise dominates on account of poor electromagnetic coupling. The SFTD method can suppress jitter noise, which makes it greatly competitive with the traditional method of RTD fluxgate, as well as the traditional harmonic fluxgates, in terms of sensitivity and noise performance. Moreover, when a small length–diameter ratio magnetic core is utilized for miniature fluxgates, the increasing demagnetizing coefficient will be the main problem as a consequence of the increasing magnetic noise, which would decrease the signal–noise ratio of the induced signal. The SFTD method can optimize the noise performance of the RTD fluxgate sensor and provides an efficient way of further development for the RTD fluxgate sensor.

However, the sampling rate should be increased for high time resolution and small noise level, which may cause another problem on data size. The possible solution is to utilize a DAQ with a trigger condition to reduce the data size significantly, through this way RTD sensors with SFTD method may work like traditional fluxgates. Therefore, the comparative results in two different methods should be the focus of this paper.
